# Large-scale school scoliosis screening in a multi-ethnic, high-altitude region of southwestern China: an epidemiological study of 69,811 children and adolescents

**DOI:** 10.3389/fpubh.2025.1659046

**Published:** 2025-10-24

**Authors:** Shanshan Song, Ying He, Qing Zhen, Tiejun Shui, Weiwei An, Limei Dai, Min Tan, Jiaxin Hao, Ruifang Song, Yong Shen, Xiangyu Yan, Litao Chang

**Affiliations:** ^1^State Key Laboratory for Diagnosis and Treatment of Severe Zoonotic Infectious Diseases, Key Laboratory for Zoonoses Research of the Ministry of Education, Department of Epidemiology and Biostatistics, School of Public Health, Jilin University, Changchun, Jilin, China; ^2^The People's Hospital of Lincang, Lincang, Yunnan, China; ^3^Yunnan Center for Disease Control and Prevention, Kunming, Yunnan, China; ^4^School of Disaster and Emergency Medicine, Tianjin University, Tianjin, China

**Keywords:** scoliosis, adolescents, epidemiology, high-altitude, ethnic minorities

## Abstract

**Objectives:**

To establish the first large-scale epidemiological profile of scoliosis among children and adolescents in Yunnan, China, and identify key demographic, anthropometric, and geographic determinants in this multi-ethnic, high-altitude region.

**Study design:**

A population-based cross-sectional screening survey using multistage stratified cluster sampling.

**Methods:**

A population-based cross-sectional survey of 69,811 students aged 6–18 was conducted in 2021 using multistage stratified cluster sampling across Yunnan, which included demographic information (gender, ethnicity, grade), anthropometric measurements (height, weight), and scoliosis outcomes (presence and severity assessed via scoliometer-based Angle of Trunk Rotation). Regional covariates (altitude, economic status) were also incorporated. Multi-factor regression analysis identified risk factors.

**Results:**

Initial screening identified 1,379 scoliosis cases, yielding an overall detection rate of 1.98%. The rate was significantly higher in females than in males (2.4% vs. 1.6%, *p* < 0.001), with males showing higher rates at ages 14 and 16, and females exhibiting consistently higher detection during early and mid-adolescence and a peak at age 17 (3.7%). The detection rate also increased progressively with education level (primary school: 1.0%; middle school: 2.7%; high school: 3.3%). Multivariable regression analysis indicated that scoliosis risk was positively associated with female sex (*AOR* = 1.738, 95% *CI*: 1.546–1.954), higher education levels (middle school: *AOR* = 1.703, 95% *CI*: 1.408–2.059; high school: *AOR* = 1.891, 95% *CI*: 1.534–2.331), and greater height (per cm *AOR* = 1.060, 95% *CI*: 1.052–1.069). Protective factors included rural residence (*AOR* = 0.717, 95% *CI*: 0.638–0.805), minority ethnicity (*AOR* = 0.856, 95% *CI*: 0.760–0.964), moderate (*AOR* = 0.515, 95% *CI*: 0.452–0.586) or low GDP (*AOR* = 0.067, 95% *CI*: 0.044–0.104), medium (*AOR* = 0.075, 95% *CI*: 0.049–0.116) or high altitude (*AOR* = 0.098, 95% *CI*: 0.068–0.140), and greater weight (*AOR* = 0.955, 95% *CI*: 0.947–0.962).

**Conclusion:**

The elevated detection rate in Yunnan highlights the influence of regional sociodemographic and environmental factors. Findings support tailored screening strategies, particularly for urban female adolescents and minority populations in high-altitude areas.

## Introduction

Spinal curvature abnormalities mainly include scoliosis and kyphosis, with scoliosis being the most common form (1). Scoliosis is a three-dimensional deformity characterized by lateral curvature and vertebral rotation in one or more spinal segments (2). According to the criteria of the Scoliosis Research Society (SRS), scoliosis is diagnosed when the Cobb angle measured from a standing anteroposterior X-ray exceeds 10° (3).

Idiopathic scoliosis (IS) accounts for 80% of all cases, with adolescent idiopathic scoliosis (AIS) comprising the vast majority (80%) of these cases. The term “idiopathic” indicates that its etiology remains unclear (4). AIS shows significant regional variations in prevalence, with rates reported as follows: 1–3% in the United States (3), 5.2% in Germany (5), and 0.4–2.5% in Asia (6). Scoliosis remains highly prevalent in China, with notable regional differences (7, 8). A 2021 meta-analysis of data from 2 million primary and secondary school students revealed a primary screening positivity rate of 4.40% and a diagnostic rate of 1.23% for scoliosis in mainland China (9). In recent years, the incidence has continued to rise (10). Notably, adolescents, particularly those aged 11 and older, represent a high-risk group for scoliosis (11). Scoliosis has become the third most significant health issue affecting children and adolescents, following myopia and obesity (12). If left untreated, early symptoms may remain hidden, and untreated scoliosis can progressively develop into bodily deformity, leading to potential damage to pulmonary and neurological functions, and even psychological disorders (3, 13). Some patients also experience comorbidities that exacerbate the disease burden. Therefore, early screening and targeted interventions for children and adolescents are critical in delaying disease progression, reducing surgical risks, and easing the economic burden (14).

Scoliosis screening programs originated in the 1960s in the United States (15), while China began its scoliosis screening initiatives in 1995 in Hong Kong (16). Since then, mainland cities such as Beijing and Guangzhou have followed suit in implementing adolescent scoliosis screening programs (17). Both domestic and international scholars generally agree that adolescents are the primary focus for scoliosis screening and intervention (15). Yunnan Province, located in the southwestern frontier of China, is characterized by complex geography, diverse ethnicities, dispersed populations, and unequal medical resources, which have led to a late start and substantial challenges in scoliosis screening efforts (18). Existing research has mainly focused on major cities, with a lack of large-scale epidemiological data from southwestern regions and areas inhabited by ethnic minorities (9). Conducting scoliosis screening research in this region will help assess the spinal health status of children and adolescents in non-central urban areas and provide scientific evidence for regional prevention and control strategies. This study, based on common disease monitoring data from 32 counties in Yunnan Province in 2021, aims to evaluate the initial screening detection rate of scoliosis among primary and secondary school students, analyze the distribution characteristics of scoliosis patients, and explore influencing factors in children and adolescents.

## Methods

### Study design

This large-scale scoliosis screening study analyzed 2021 surveillance data from Yunnan Province, a mountainous frontier region in southwestern China with an average elevation of 1,980 meters and diverse subtropical to tropical climates. As a key hub within the China-Myanmar-Laos Economic Corridor and the Greater Mekong Subregion, Yunnan’s distinct geographical and socioeconomic profile offers valuable insights for other middle-income mountainous areas.

Data were obtained from the Yunnan Province for 2021 were obtained from the Yunnan Provincial Center for Disease Control and Prevention, covering 16 prefecture-level cities. Using a stratified sampling approach based on the *2021 National Technical Manual for Surveillance and Intervention on Common Diseases and Health Determinants in Students*, we selected at least one urban and one suburban/rural site per city. Urban regions contributed seven randomly selected schools (two primary, two junior high, and three senior high schools), while suburban/rural regions contributed five (two primary, two junior high, and one senior high school). Within each school, cluster sampling was performed with grade as the stratification variable and class as the sampling unit, ensuring at least 80 participants per grade. The final analytic sample comprised 69,811 students across 32 counties or districts. The study protocol was approved by the Medical Ethics Committee of the Yunnan Provincial Center for Disease Control and Prevention (Approval No. 2025-8).

### Data collection and physical examination

Data were collected in accordance with the above manual using the Student Key Common Diseases Detection Form (Primary and Secondary School Edition), which included general demographic characteristics (screening site, grade, gender, ethnicity, date of birth, date of physical examination, height, weight, etc.), as well as outcome indicators for scoliosis detection (presence, type, and severity).

Altitude data were obtained from a high-precision digital elevation model (DEM) provided by the Institute of Geographic Sciences and Natural Resources Research, Chinese Academy of Sciences. The median altitude for each monitoring site was extracted using ArcGIS software (version 18.0). Gross domestic product (GDP) data were retrieved from the Yunnan Statistical Yearbook 2021, with county-level per capita GDP serving as the economic indicator. Countries were divided into high, medium, and low groups (tertiles) based on ranked values.

**Figure 1:**
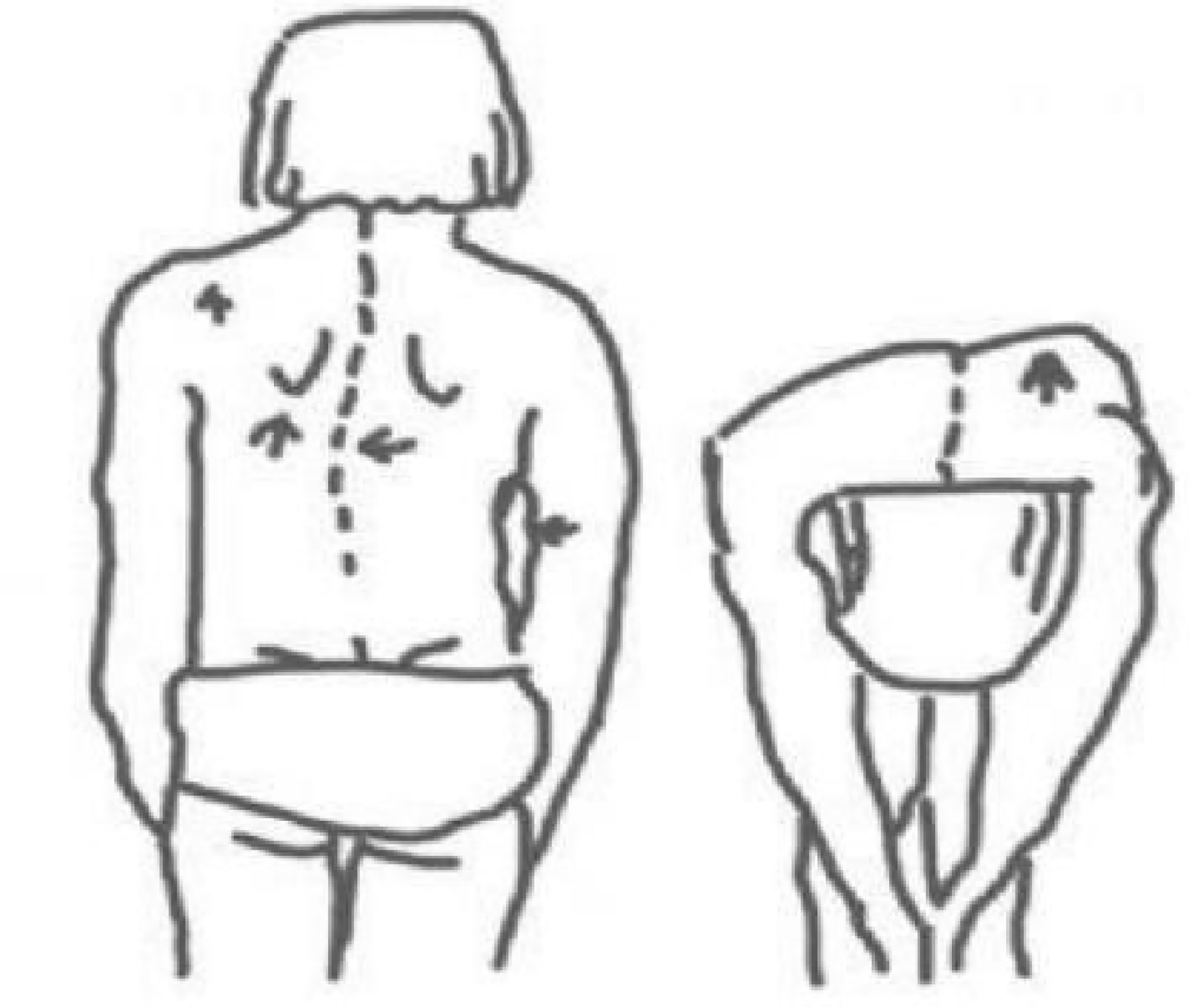
General inspection and Adams forward bending test for scoliosis.

**Figure 2:**
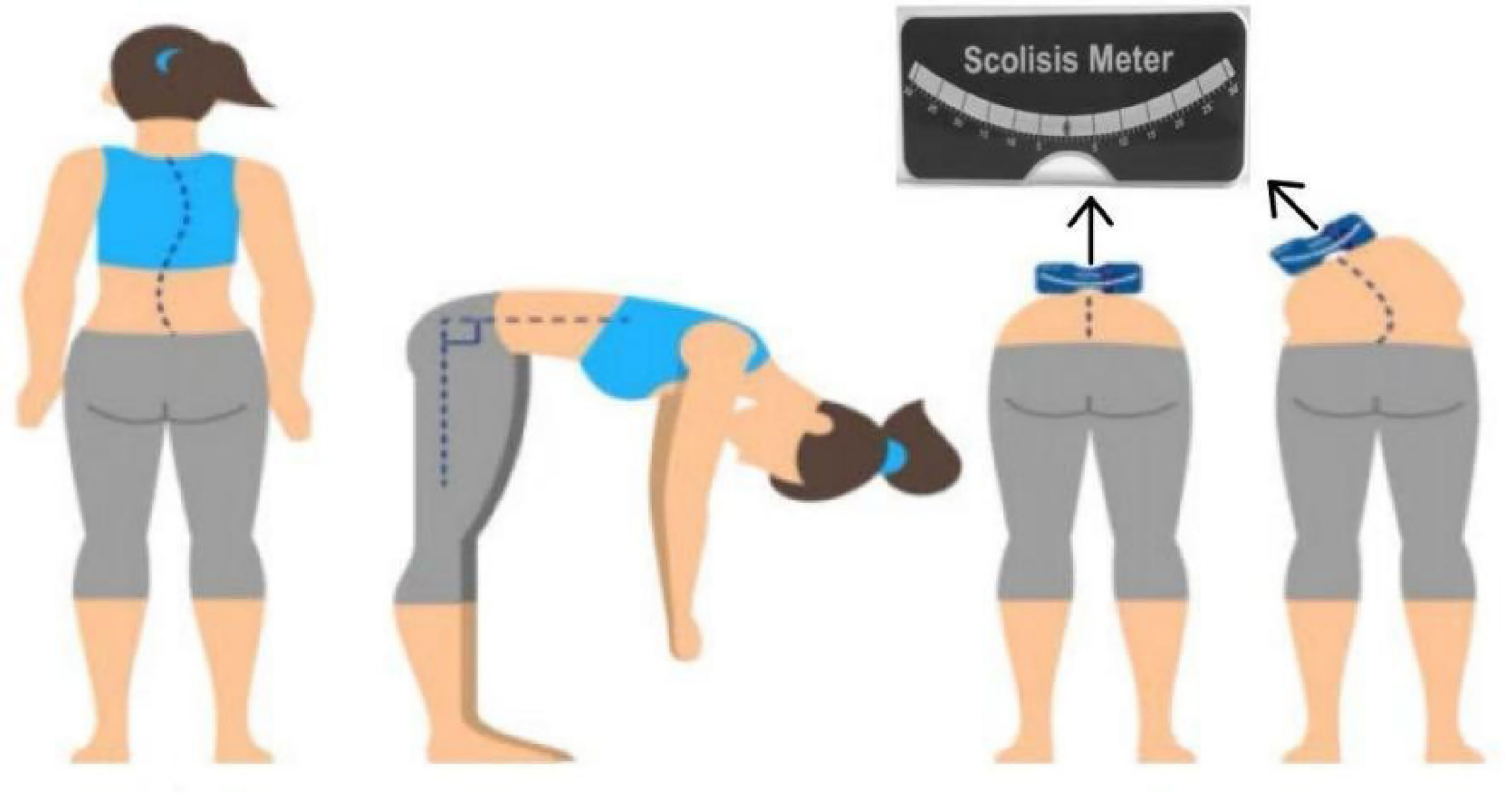
Examination using a scoliometer.

The scoliosis screening was conducted by professionally trained technicians who passed a rigorous examination. Prior to the screening, the teachers, students, and their parents were informed about the screening content, and informed consent was obtained. The screening method followed *the Technical Guidelines for the Prevention and Control of Spinal Curvature Abnormalities in Children and Adolescents* (GB/T 16133-2014, China’s recommended national standard for medical practice) (19), and the procedures were as follows: ① General inspection: The subject was asked to remove their shoes and stand naturally. For males, the upper body was left exposed; for females, only undergarments were worn. The examiner observed from behind whether both shoulders were level, scapulae were symmetrical, the lumbar curve was even, iliac crests were at the same level, and there was no deviation in the line of the spinous processes (Figure 1). ② The examinee stood with their back to the examiner, knees straight and ankles together. With arms extended and palms together, the trunk was slowly bent forward to approximately 90°. The hands were placed between the knees to avoid compensatory shifts. The examiner viewed the back horizontally from thoracic to lumbar regions, checking for symmetry along both sides of the spine. The test was considered positive if a unilateral rib hump, paraspinal asymmetry, or muscular prominence was observed, indicating a high suspicion of scoliosis. ③ Angle of trunk rotation (ATR) measurement: While maintaining the forward-bending position, a scoliometer was used to measure the ATR sequentially in the thoracic, thoracolumbar, and lumbar regions. The maximum ATR value and its location were recorded. An ATR ≥ 5°at the most prominent asymmetric site suggested a high likelihood of scoliosis. This threshold effectively identifies most cases of scoliosis with a Cobb angle ≥ 10°during screening. ④ Spinal movement test: Individuals with suspected abnormalities in the general inspection but a negative forward bending test performed spinal flexion, extension, left and right lateral bending, and left and right axial rotation twice each. The examiner then rechecked the spine for any residual curvature (Figure 2).

**Figure 3:**
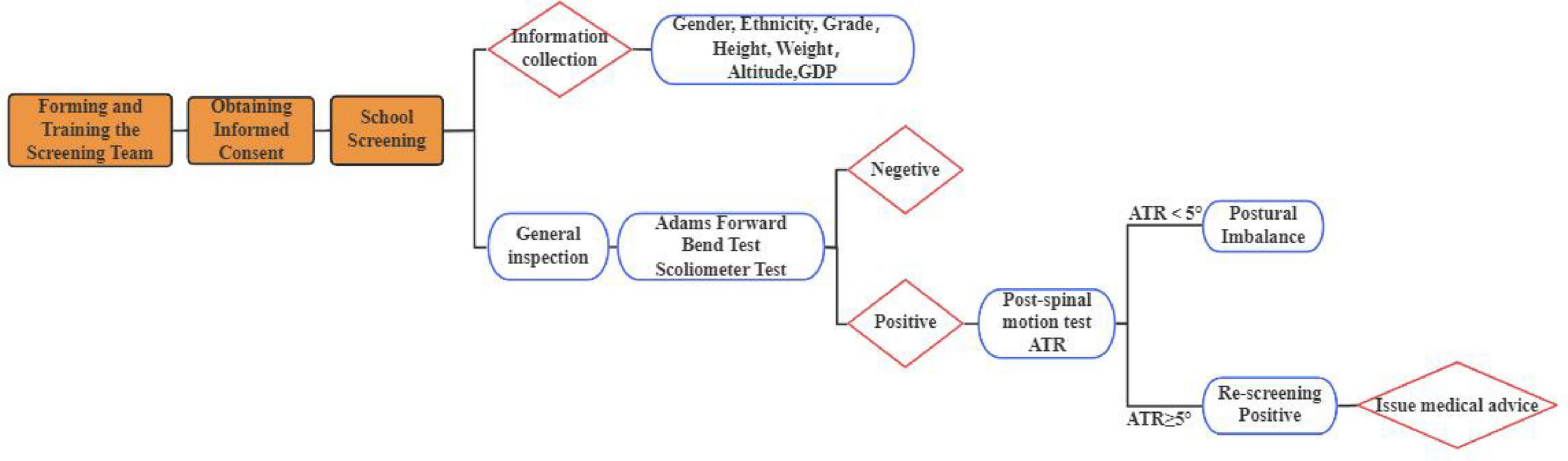
Spinal scoliosis school screening process.

Scoliosis screening outcomes were classified as follows: No Scoliosis (No abnormalities on general inspection and forward bending test, and a static ATR < 5°); Postural Inbalance (Abnormal general inspection, positive forward bending test, or static ATR ≥ 5°, but with ATR < 5° after the movement test); Positive Screening (Any one of the following: abnormal general inspection, positive forward bending test, or static ATR ≥ 5°, with ATR remaining ≥5° after the movement test). For analytical purposes, the “No Scoliosis” and “Postural Inbalance” groups were collectively classified as screening-negative, while the “Positive Screening” group was classified as screening-positive. Students with positive screening results were strongly referred to medical institutions for radiographic examination (Cobb angle measurement) to confirm diagnosis (Figure 3).

## Results

### Basic characteristics

**Figure 4:**
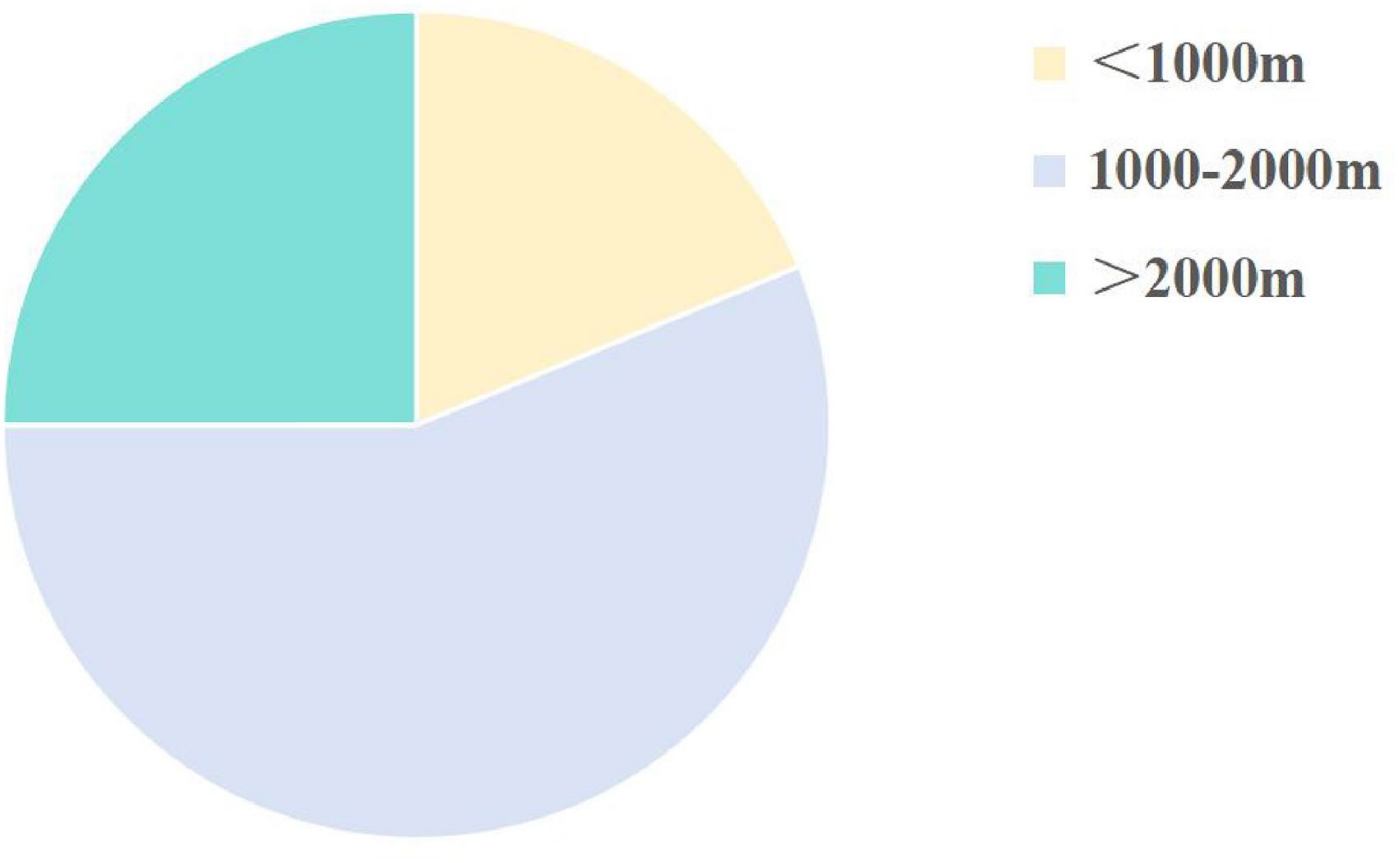
Distribution of the scoliosis screening population across altitude groups.

A total of 69,811 students aged 6–18 years were screened in this study, including 34,472 males (49.38%) and 35,339 females (50.62%). Regarding regional distribution, more than half of the students were from urban areas (55.57%, 38,792 students), while 44.43% (31,019 students) were from rural areas. Primary school students comprised the majority of the study population (51.27%, 35,793 students), followed by middle school students (24.82%, 17,329 students) and high school students (23.91%, 16,689 students). Ethnic distribution was diverse, with Han students representing 52.92% (36,947 students) and minority ethnic students accounting for 47.08% (32,864 students). Additionally, the study population was widely distributed across various geographic altitudes and levels of economic development. Most students lived in regions with altitudes ranging from 1,000 to 2000 meters (62.09%, 43,348 students, Figure 4), and were primarily concentrated in areas with moderate economic development (43.19%, 30,148 students). Detailed data can be found in Table 1.

**Table 1:** Demographic characteristics of the screened population (*N* = 69,811).

Variable	No. of students	Age (years)	Boy (*N* = 34,472) *n* (%)	Girl (*N* = 35,339) *n* (%)	Chi- square	*P*
Education level						
Primary school	35,793	8.79 ± 1.81	18,284 (53.04)	17,509 (49.55)	113.697	<0.001
Junior high school	17,329	13.40 ± 1.03	8,492 (24.63)	8,837 (25.01)		
Senior high school	16,689	16.42 ± 1.01	7,696 (22.33)	8,993 (25.45)		
Screening site						
Urban	38,792	12.23 ± 3.64	19,135 (55.5)	19,657 (55.6)	0.094	0.759
Rural	31,019	11.17 ± 3.32	15,337 (44.5)	15,682 (44.4)		
Ethnicity						
Han	36,947	11.61 ± 3.52	18,399 (49.8)	18,548 (52.5)	5.521	0.019
Minority	32,864	11.93 ± 3.56	16,073 (50.2)	16,791 (47.5)		
Gross Domestic Product (GDP)						
High	17,461	11.65 ± 3.53	8,735 (25.3)	8,726 (24.7)	3.979	0.137
Medium	30,148	11.80 ± 3.55	14,838 (43.0)	15,310 (43.3)		
Undevelopment	22,202	11.80 ± 3.55	10,899 (31.6)	11,303 (32.0)		
Altitude						
<1,000	9,542	11.78 ± 3.51	4,744 (13.8)	4,798 (13.6)	20.297	<0.001
1,000–2000	43,348	11.75 ± 3.54	21,627 (62.7)	21,721 (61.5)		
>2000	16,921	11.78 ± 3.57	8,101 (23.5)	8,820 (25.0)		

Percentages may not sum to 100.0% due to rounding.

### Detection rate

A total of 1,379 positive cases were identified in this scoliosis screening, resulting in an overall detection rate of 1.98%. A significant sex disparity was observed, with a markedly higher detection rate among female students compared to their male counterparts (2.4% vs. 1.6%; *p* < 0.001; Table 2).

**Table 2:** Demographic characteristics of scoliosis screening positive and negative.

Variable	Total (*N* = 69,811)	Screening-positive (*N* = 1,379) *n* (1.98%)	Screening-negative (*N* = 68,432) *n* (98.02%)	Detection rate (%)	*t* value/Chi-square value	*P*
Gender					53.092	<0.001
Male	34,472 (49.4)	547 (39.7)	33,925 (49.57)	1.6		
Female	35,339 (50.6)	832 (60.3)	34,507 (50.43)	2.4		
Height (mean + SD, cm)	147.11 ± 16.93	155.45 ± 14.74	146.94 ± 16.93		−18.518	<0.001
Weight (mean + SD, kg)	42.45 ± 15.81	46.72 ± 13.38	42.36 ± 15.85		−10.136	<0.001
BMI (mean + SD, kg/m^2^)	18.90 ± 4.05	18.90 ± 3.23	18.90 ± 4.07		−0.026	0.979

**Figure 5:**
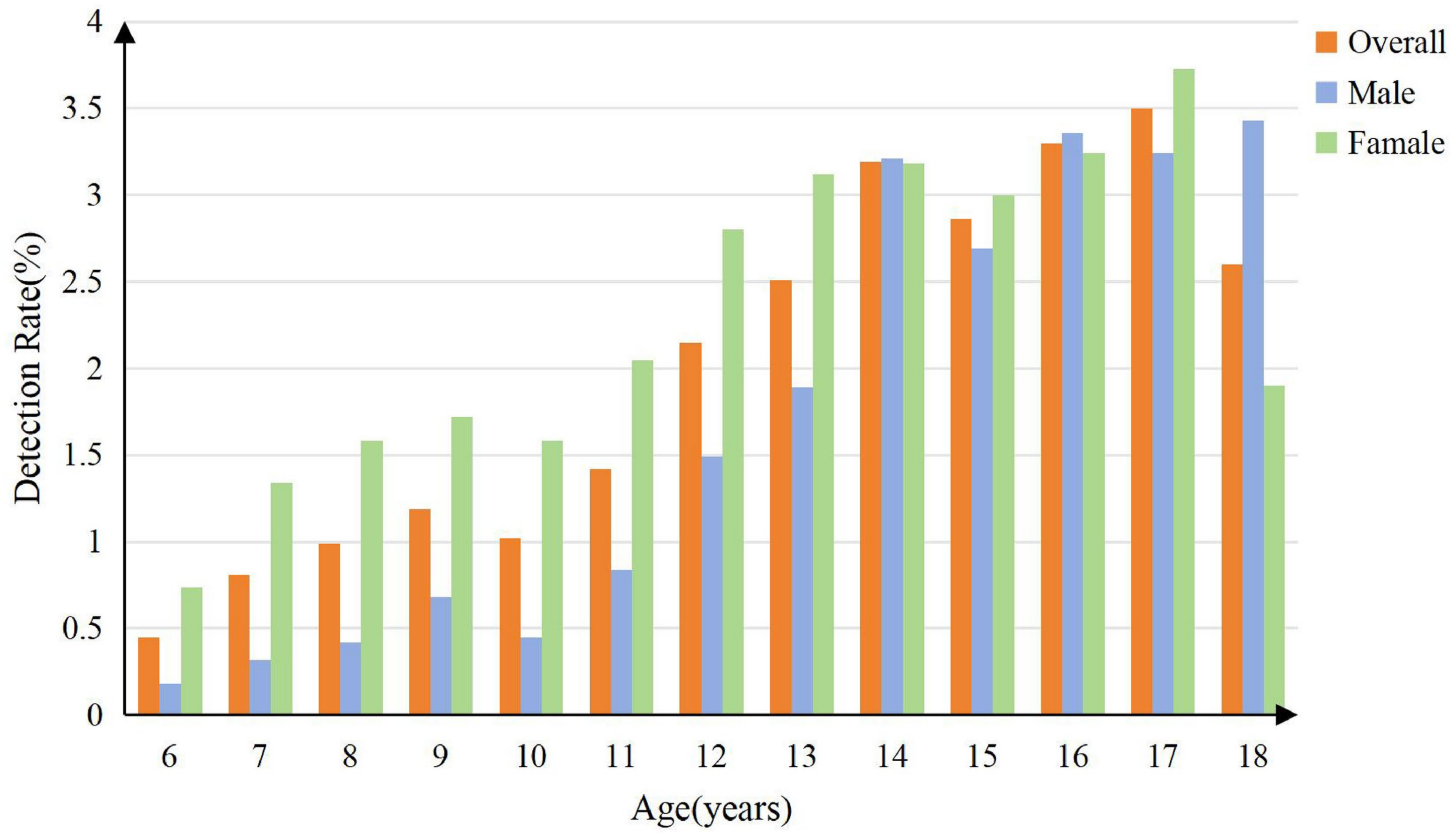
Distribution of scoliosis detection rates across age and sex groups.

The disparity exhibited a dynamic pattern across age groups. As shown in Figure 5, the detection rate was consistently higher in females, particularly during early and mid-adolescence (ages 6–14, 16, and 17), while males showed a higher detection rate at ages 14, 16, and 18 years. The detection rate peaked at 17 years for females (3.7%), which was significantly higher than the peak for males (3.4%) at 18 years. A secondary peak was also observed for males at 16 years (3.4%).

Regarding anthropometric measures, screening-positive cases were significantly taller and heavier than negative cases (*p* < 0.001 for both; Table 2). However, no significant intergroup difference was found in Body Mass Index (BMI).

### Detection rates stratified by screening site, ethnicity, education level, GDP, and altitude

Scoliosis detection rates varied significantly across key demographic and geographic variables (all *p* < 0.001; Table 3). Higher detection rates were observed in urban areas (2.4%), the Han ethnic group (2.4%), senior high school students (3.3%), high-GDP regions (3.2%), and areas with altitude below 2000 meters (2.3–2.7%). The lowest rate was observed in regions above 2000 meters (0.7%).

**Table 3:** Scoliosis detection rates among students stratified by ethnicity, education level, and GDP.

Variable	Total (*N* = 69,811)	Screening- positive (*N* = 1,379) *n* (1.98%)	Screening- negative (*N* = 68,432) *n* (98.02%)	Detection rate (%)	*t* value/Chi-square value	*P*
Ethnicity					79.058	<0.001
Han	36,947 (52.9)	893 (64.8)	36,054 (52.7)	2.4		
Minority	32,864 (47.1)	486 (35.2)	32,378 (47.3)	1.5		
Education level					378.972	<0.001
Primary school	35,793 (51.3)	357 (25.9)	35,436 (51.8)	1.0		
Junior high school	17,329 (24.8)	469 (34.0)	16,860 (24.6)	2.7		
Senior high school	16,689 (23.9)	553 (40.1)	16,136 (23.6)	3.3		
Gross Domestic Product (GDP)					190.954	<0.001
High	17,461 (25.0)	558 (40.5)	16,903 (24.7)	3.2		
Medium	30,148 (43.2)	527 (38.2)	29,621 (43.3)	1.7		
Undevelopment	22,202 (31.8)	294 (21.3)	21,908 (32.0)	1.3		

### Detection rates stratified by screening sites, altitude, and counties

**Figure 6:**
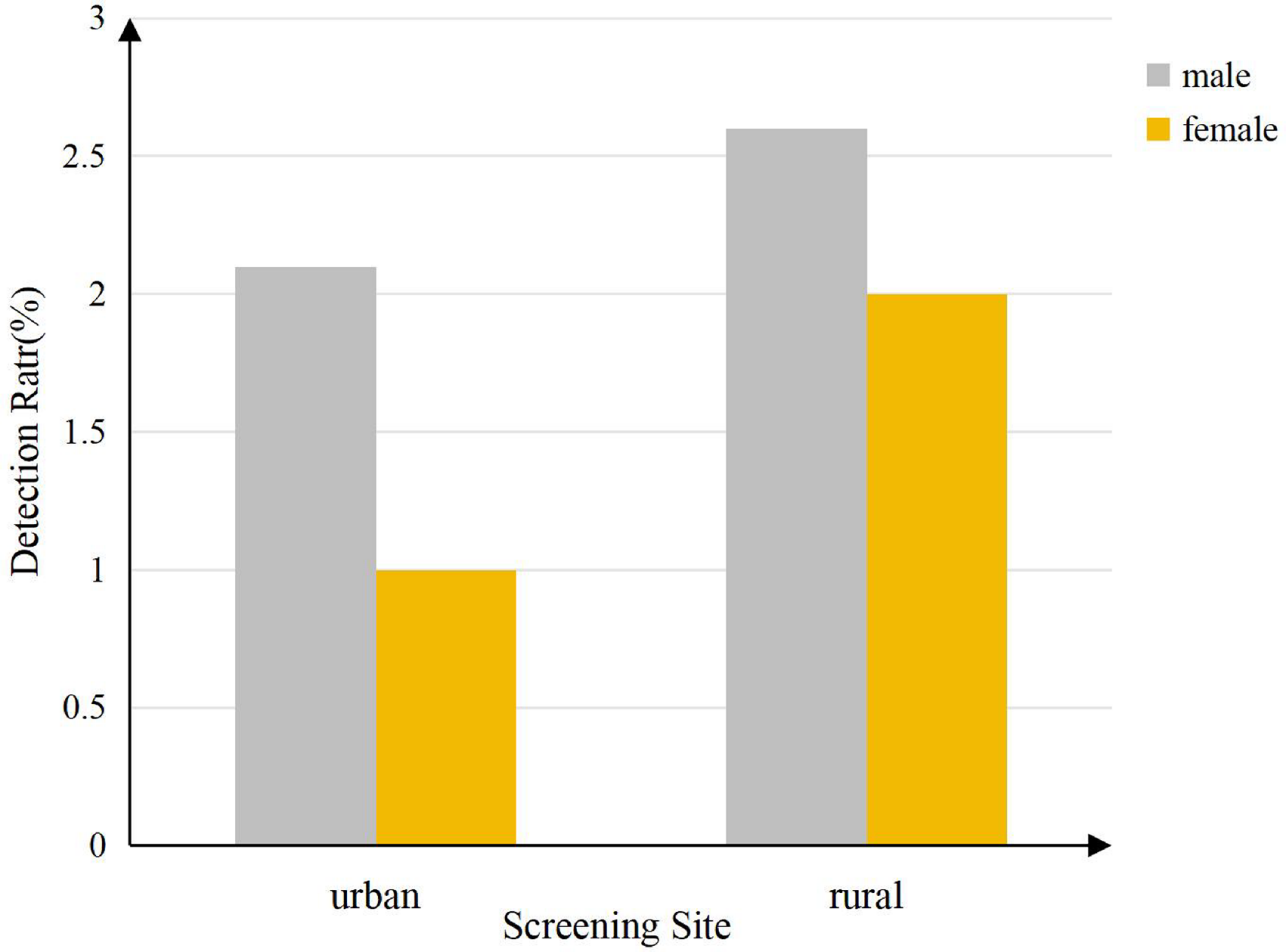
Scoliosis detection rate by gender and screening site.

**Figure 7:**
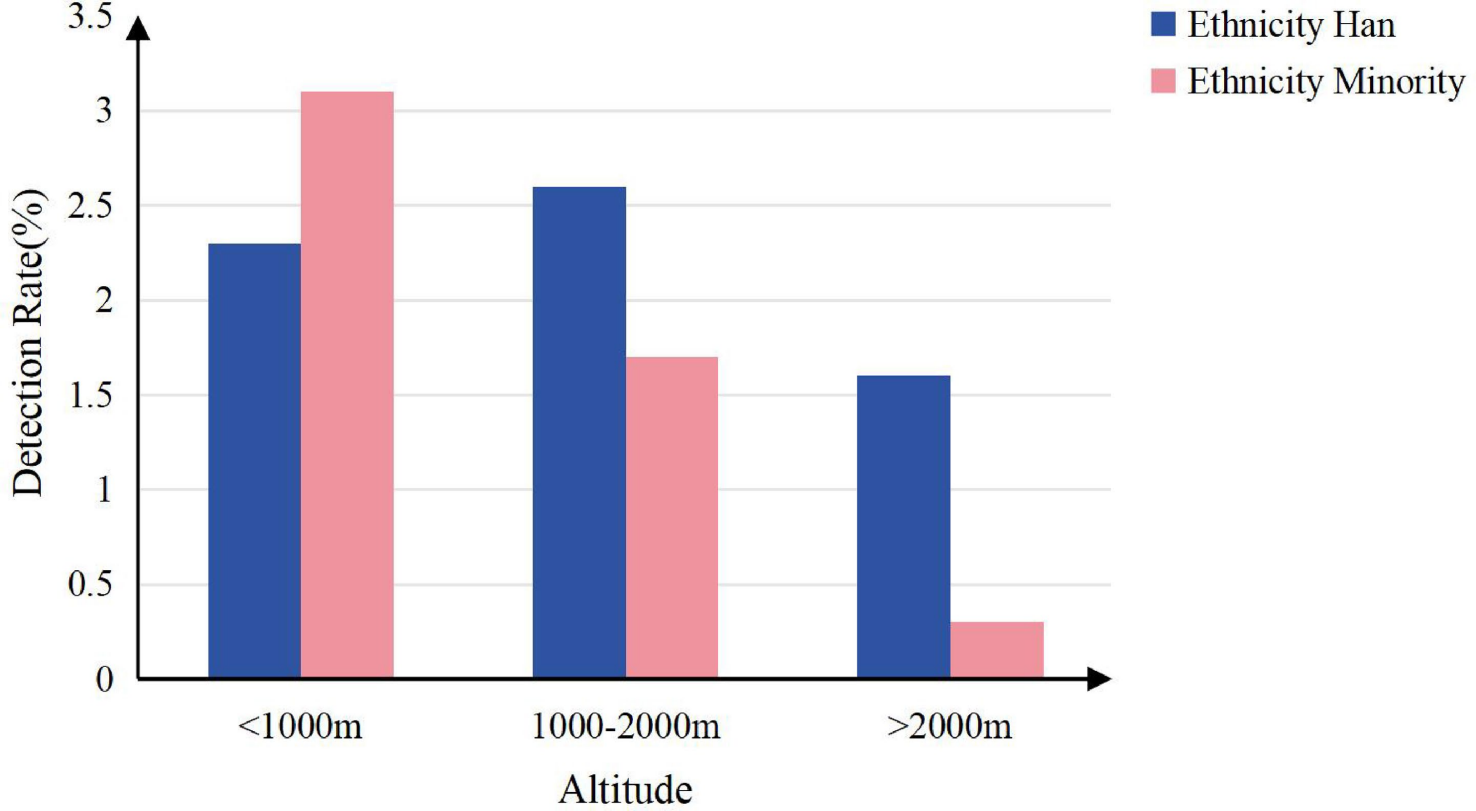
Scoliosis detection rate by ethnicity and altitude region.

Scoliosis detection rates differed significantly by screening site, altitude group (*p* < 0.001; Figures 6, 7), with higher rates observed in urban areas (2.4% vs. 1.5% rural) and lower altitudes (2.7% at <1,000 m vs. 0.7% at >2000 m), and varied widely across counties (range: 0.1–7.4%; Table 4).

**Table 4:** Scoliosis detection rates among students stratified by screening site, altitude and country.

Variable	Total (*N* = 69,811)	Screening-positive (*N* = 1,379) *n* (1.98%)	Screening-negative (*N* = 68,432) *n* (98.02%)	Detection rate (%)	*P*
Screening Site					<0.001
Urban	38,792	913	37,879	2.4	
Rural	31,019 (44.4)	466	30,553	1.5	
Altitude					<0.001
<1,000	9,542	260	9,282 (13.6)	2.7	
1,000–2000	43,348	993	42,355 (61.9)	2.3	
>2000	16,921	126	16,795 (24.5)	0.7	
Country					<0.001
Kunming	4,321	318	4,003	7.4	
Zhaotong	4,261	92	4,169	2.2	
Qujing	4,769	90	4,679	1.9	
Chuxiong	4,663	69	4,594	1.5	
Yuxi	4,325	131	4,194	3.0	
Honghe	4,046	19	4,027	0.5	
Wenshan	3,985	22	3,963	0.6	
Puer	3,898	131	3,767	3.4	
Banna	4,868	206	4,662	4.2	
Dali	4,500	81	4,419	1.8	
Baoshan	4,065	118	3,947	2.9	
Dehong	4,674	54	4,620	1.2	
Lijiang	4,461	14	4,447	0.3	
Nujiang	3,792	15	3,777	0.4	
Diqing	4,407	5	4,402	0.1	
Lincang	4,776	14	4,762	0.3	

### Correlation analysis and variance inflation factor assessment

A two-step approach was used to assess and address potential multicollinearity among the independent variables before constructing the multi-factor regression model. The variables of interest included Gender, Height, Weight, BMI, Ethnicity, Education Level, Screening Site, GDP and Altitude.

**Figure 8:**
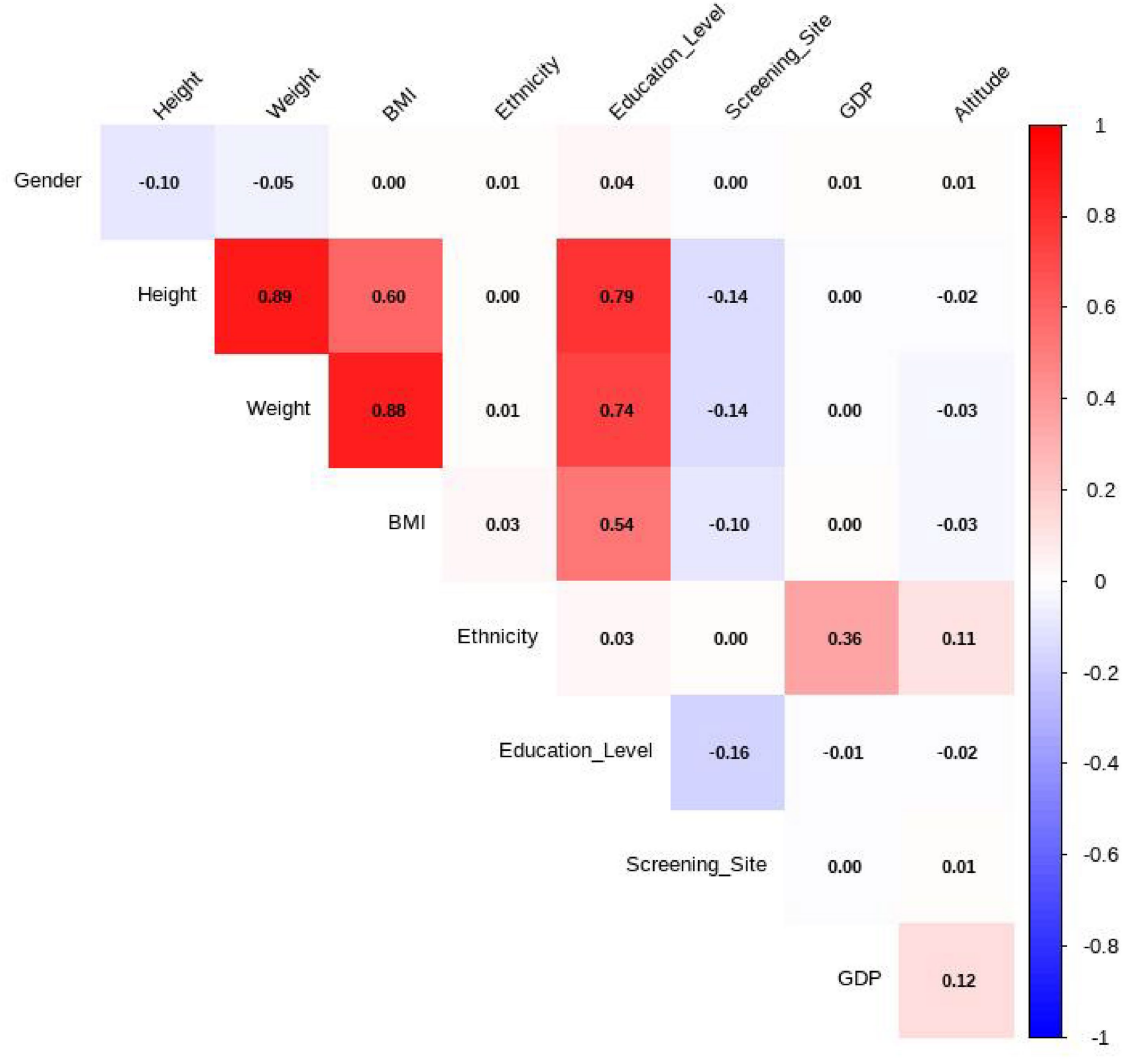
Spearman correlation matrix of the variables.

First, Spearman correlation analysis was conducted (Figure 8). A strong correlation was identified between BMI and both height (*ρ* = 0.89) and weight (ρ = 0.88). Due to its strong collinearity with these anthropometric measures and to avoid model instability, BMI was excluded from subsequent analyses based on a pre-defined correlation coefficient threshold of |ρ| > 0.8. Next, variance inflation factors (VIFs) were calculated for the remaining variables to further assess multicollinearity. After excluding BMI, all variables had VIF values well below the conservative threshold of 5, indicating that severe multicollinearity was not a concern for the final multivariate model.

### Multi-factor regression analysis and factors associated with scoliosis

Multi-factor regression analyses identified multiple factors associated with scoliosis (Table 5). Female participants had significantly higher odds of scoliosis than males (*OR =* 1.50, 95% *CI*: 1.34–1.67). Height (*OR* ***=*** 1.03 per cm, 95% *CI*: 1.03–1.04) and weight (*OR* = 1.02 per kg, 95%*CI*: 1.01–1.02) were both positively associated with scoliosis. Participants from minority ethnic groups had lower odds compared to the Han majority (*OR =* 0.61, 95% *CI*: 0.54–0.68). Educational level exhibited a strong gradient: using primary school as the reference, the odds were significantly higher for middle school (*OR* = 2.76, 95% *CI*: 2.40–3.17) and high school students (*OR* = 3.40, 95% *CI*: 2.97–3.89) compared to those with primary school education. Screenings conducted in rural areas (*OR* = 0.63, 95%*CI*: 0.57–0.71), and in regions with medium (*OR* = 0.54, 95%*CI*: 0.48–0.61) or low GDP (*OR* = 0.41, 95% *CI*: 0.35–0.47), were associated with reduced odds. Living at higher altitudes (1000–2000 m: *OR* = 0.84, 95% *CI*: 0.73–0.96; >2000 m: *OR* = 0.27, 95% *CI*: 0.22–0.33) appeared to offer a protective effect.

**Table 5:** Multi-factor regression analysis of factors associated with scoliosis in the unadjusted model and the adjusted model (*N* = 69,811).

Variable	Unadjusted *OR* (95% CI)	*P*	Adjusted *OR* (95% CI)	*P*
Gender				
Male	1		1	
Female	1.495 (1.341, 1.667)	<0.001	1.738 (1.546, 1.954)	<0.001
Height	1.033 (1.030, 1.037)	<0.001	1.060 (1.052, 1.069)	<0.001
Weight	1.016 (1.013, 1.019)	<0.001	0.955 (0.947, 0.962)	<0.001
BMI	1.000 (0.987, 1.013)	0.979		
Ethnicity				
Han	1		1	
Minority	0.606 (0.542, 0.678)	<0.001	0.856 (0.760, 0.964)	0.011
Educational level				
Primary school	1		1	
Middle school	2.761 (2.403, 3.173)	<0.001	1.703 (1.408, 2.059)	0.001
High school	3.402 (2.974, 3.891)	<0.001	1.891 (1.534, 2.331)	0.001
Screening site				
Urban	1		1	
Rural	0.633 (0.565, 0.708)	<0.001	0.717 (0.638, 0.805)	<0.001
Gross Domestic Product (GDP)				
High	1		1	
Medium	0.539 (0.478, 0.608)	<0.001	0.515 (0.452, 0.586)	<0.001
Low	0.407 (0.352, 0.469)	<0.001	0.067 (0.044, 0.104)	<0.001
Altitude				
<1,000 m	1		1	
1,000-2000 m	0.837 (0.729, 0.961)	0.012	0.075 (0.049, 0.116)	<0.001
>2000 m	0.268 (0.216, 0.332)	<0.001	0.098 (0.068, 0.140)	<0.001

OR, odds ratio; CI, confidence interval. All variables shown were included in the multivariable logistic regression model.

To address multicollinearity (VIF > 10), BMI was excluded from the final adjusted model. The model revealed several notable findings: the risk for female gender increased (*AOR =* 1.74, 95% *CI*: 1.55–1.95), while the effect of height became more pronounced (*AOR =* 1.06 per cm, 95% *CI*: 1.05–1.07). Conversely, weight exhibited a significant protective effect (*AOR =* 0.96 per kg, 95% *CI*: 0.95–0.96). The protective association for minority ethnicity remained significant (*AOR =* 0.86, 95% *CI*: 0.76–0.96). Particularly robust were the protective effects of low GDP (*AOR =* 0.07, 95% *CI*: 0.04–0.10) and high altitudes.

## Discussion

### Prevalence of scoliosis in Yunnan Province

The global prevalence of adolescent idiopathic scoliosis (AIS) is approximately 1.34% (20), with significant regional variations. In the United States, the prevalence of AIS among individuals aged 10–16 years ranges from 1 to 3% (3), while in Germany, it is as high as 5.2% (5). In Asia, the prevalence varies between 0.4 and 2.5% (6), and in mainland China, the overall prevalence among primary and secondary school students was reported to be 1.23% in 2021 (9). In this study, the detection rate in Yunnan Province was 1.98%, which is higher than the global average (1.34%) and the national average in China (1.23%), but lower than the rates reported in central and coastal metropolitan areas such as Taiyuan (3.1%) and Guangzhou (8.2%) (21). These differences may be attributed to variations in screening criteria, ethnic disparities, economic development levels, and lifestyle factors.

### Gender, age, and scoliosis

The results of this study revealed that the detection rate of scoliosis was significantly higher in females aged 6–14, 16, and 17 years compared to males, with the highest rate observed at 17 years before it declining at 18 years. In contrast, males exhibited slightly higher detection rates than females at 14 and 16, with a significant peak at 18 years. From age 11 onward, the detection rate in females consistently surpassed that in males, but it tended to stabilize or gradually decline with age, consistent with findings from previous studies (9, 22, 23). Adolescents, particularly those aged 11–18 years, represent a high-risk group for scoliosis (24). Scoliosis typically develops during the pre-pubertal stage and progresses rapidly throughout adolescence, resulting in markedly higher detection rates in adolescents compared to children under 11 years of age (25). The incidence of adolescent idiopathic scoliosis (AIS) is higher in females than in males (26), with an earlier peak onset age (13 years) in females, potentially due to factors such as pubertal development, estrogen fluctuations, and changes in bilateral spinal muscle tension (9). A study on Turkish AIS patients further revealed abnormal growth patterns with significant sexual dimorphism during puberty: female patients exhibited higher BMI pre- and during the growth spurt, whereas males showed lower BMI at pubertal stage 5. This suggests that distinct pathophysiological processes may underlie AIS in males and females, which could partially explain the gender-specific and age-stage-specific (e.g., mid-to-late puberty) risk elevations observed in the present study (27). Estrogen plays a critical role in the development and progression of AIS by abnormally modulating bone metabolism, affecting spinal biomechanical stability, and influencing the paraspinal muscle system through receptor-mediated mechanisms (28). Additionally, abnormal leptin bioavailability may contribute to reduced BMI and increased AIS risk in adolescent females (29).

### Body development and scoliosis

Epidemiological surveys show that the prevalence of spinal curvature abnormalities increases significantly with educational level. High school students have approximately 1.9 times the risk of suspected scoliosis compared to primary school students (*OR* = 1.891, 95% CI: 1.534–2.331). The incidence of suspected scoliosis peaks at age 17 (overall: 3.50%; females: 3.73%), which is about 2 years later than the previously reported peak at age 15 (30). This difference may be attributed to variations in study design, such as using skeletal maturity instead of chronological age for staging, as well as modifiable risk factors, including academic pressure, sedentary behavior, and insufficient physical activity, all of which may affect the age distribution of scoliosis (31, 32). These three factors, particularly in high school, often occur together: increased academic burden leads to prolonged periods of fixed postures (such as sitting and studying), while physical activity significantly decreases. The stability and physiological curvature of the spine depend on the support from surrounding muscles and ligaments (33). A lack of exercise and poor lifestyle habits, such as insufficient care for the back, can result in reduced bone density, poor skeletal development, and weakened muscles and ligaments, making it difficult to maintain normal spinal curvature (34). This is supported by clinical research that further confirms adolescents with moderate scoliosis (Cobb angle >20°) exhibit significantly lower bone mineral density and vitamin D deficiency compared to their peers with mild or no scoliosis (35). Moreover, Chow et al.’s (36) comparative study on the effects of different backpack loads (0, 7.5, 10, 12.5, and 15% BW) on scoliosis indicated that improper backpack carriage methods (37, 38) and excessive load increase asymmetric spinal loading, impair postural balance, and thereby elevate the risk of muscle fatigue and scoliosis progression. A longitudinal study in the UK involving 4,640 participants suggested that, after adjusting for confounding factors such as gender and age, children who engaged in moderate-to-high-intensity physical activity had a 30% lower risk of developing scoliosis (31, 39). Therefore, early posture education and promoting regular physical activity during the primary school years, when behavioral habits are more malleable, are crucial.

This study found that primary and secondary school students with lower body weight and taller height had a higher detection rate of scoliosis, consistent with the findings of Qui et al. (40). Spinal abnormalities in students with lower body weight are more easily identified, whereas in obese individuals, the accumulation of back fat may interfere with the accuracy of the Adams Forward Bend Test (FBT) and the Angle of Trunk Rotation (ATR) measurements. Fluctuations in body weight may also affect spinal stability, potentially influencing ATR readings. Nutritional levels indirectly impact ATR values by affecting height, weight, and BMI (41). Regions with higher economic development typically offer better nutritional resources and healthcare, which may accelerate height growth in adolescents. However, students in these areas are more likely to adopt sedentary lifestyles (42), characterized by reduced physical activity and increased screen time (43). The observed urban–rural disparity in detection rates (*AOR* = 0.717) may reflect multiple factors, including differences in healthcare access and lifestyle between regions. However, the specific mechanisms and impacts of these associations require further research for validation.

### Ethnic and scoliosis

This study revealed ethnic disparities in scoliosis detection rates, with a higher detection rate among the Han population relative to non-Han groups, aligning with earlier findings by Sun et al. (21). Several genetic loci, including SLC39A8 and ABO, have been established as significantly associated with adolescent idiopathic scoliosis risk in the Han Chinese population (44, 45). However, ethnic disparities in scoliosis prevalence likely arise from a combination of influences. Variations in genetic susceptibility, socioeconomic status, cultural background, lifestyle, and living environment across ethnic groups may collectively contribute to scoliosis risk (46, 47). Differences in nutritional intake and physical activity may further modulate spinal development. These hypotheses require further validation through large-scale multicenter studies and long-term follow-up to clarify the mechanistic role of ethnicity in the pathogenesis and progression of scoliosis.

### Altitude and scoliosis

Previous studies have suggested that high-altitude environments may be a risk factor for scoliosis, potentially mediated by mechanisms such as chronic hypoxia, low atmospheric pressure, and vitamin deficiencies (48, 49). In contrast, this study observed a significantly lower incidence of scoliosis among children and adolescents residing in medium- and high-altitude regions (≥1,000 meters) compared to those in low-altitude areas, suggesting a potential protective role of altitude. The following factors may help explain this discrepancy:

First, the unique geographical environment of Yunnan Province, which includes its low-latitude plateau setting, prolonged sunlight exposure, and distinctive patterns of temperature and humidity, may contribute to specific influences on spinal development. Additionally, long-term residence at high altitude may promote physiological adaptations or select for genetic traits that confer greater tolerance to hypoxic conditions, potentially reducing susceptibility to scoliosis. Second, children and adolescents in high-altitude areas tend to engage in higher levels of physical activity, which may enhance paraspinal muscle strength and spinal stability. Sufficient ultraviolet radiation also facilitates vitamin D synthesis, which supports skeletal health (35, 50). Furthermore, hypoxic conditions may stimulate the expression of angiogenic factors or modulate energy metabolism pathways, possibly offering protective effects on spinal development (51).

### Study limitations

Owing to the constraints of large-scale screening, our analysis represents only a preliminary exploration of risk factors, and some potential influences may not have been fully accounted for. Moreover, the cross-sectional design precludes causal inference and may not completely control for all confounding variables. Additionally, limited by the procedures of the screening study, we relied solely on the Adam’s Forward Bend Test (ATR) as a screening tool. This means that, since the protocol did not include subsequent confirmatory X-ray diagnosis, it could not be determine the prevalence of scoliosis or its changes over time. Future research should aim to refine screening strategies, establish standardized referral and follow-up protocols, and incorporate more robust methodologies—such as longitudinal cohorts or experimental studies—to further clarify the mechanisms of scoliosis and advance the standardization and systematization of screening programs.

## Conclusion

This study conducted scoliosis screening among 69,811 primary and junior high school students across 32 counties or districts within 16 prefecture-level cities in Yunnan Province, China. The screening aimed to assess the detection rate and identify influencing factors for scoliosis among children and adolescents in the region. Results showed an overall detection rate of 1.98%. Higher risk was associated with female gender, senior high school students, and greater height. Lower risk was observed among students in rural areas, ethnic minorities, regions with moderate or low economic development, medium and high-altitude regions, and those with higher body weight. These findings highlight the importance of enhancing scoliosis screening programs in Yunnan by focusing on high-risk groups including female adolescents, senior high school students, and urban students. Additionally, tailored interventions should be implemented to address health disparities in rural and ethnic minority communities.

## Data Availability

The raw data supporting the conclusions of this article will be made available by the authors, without undue reservation.
