# Supplementary material for: Large-scale school scoliosis screening in a multi-ethnic, high-altitude region of southwestern China: an epidemiological study of 69,811 children and adolescents
Source: Front Public Health. 2025 Oct 24;13:1659046. doi: 10.3389/fpubh.2025.1659046 (PMC12592162; doi:10.3389/fpubh.2025.1659046)

| ****Table 3: Spinal Deformity Screening Record Form**** |
| --- |
| ****Basic Information**** |
| Name: ________________  Sex: ________________ ID/Student ID: ________________  Grade: ________________  Class: ________________ School Location: ______ Province (Autonomous Region) ______ District (County, City) Schoo ______l Date of Birth: ______ Year ______ Month ______ Day  Examination Date: ______ Year ______ Month ______ Day |
| ****Spinal Deformity Screening Record**** |
| ****I.Scoliosis Screening**** |
| ****(1)General Examination**** ① Normal ② Uneven shoulders ③ Asymmetric inferior scapular angles  ④ Asymmetric waistlines ⑤ Uneven iliac crest height ⑥ Deviated or spinal process line |
| ****(2) Adam's Forward Bend Test**** Thoracic segment: ① No curvature ② Left rib prominence (right convex) ③ Right rib prominence (left convex) ATR: ____° Thoracolumbar segment: ① No curvature ② Left prominence ③ Right prominence ATR: ____° Lumbar segment: ① No curvature ② Left prominence ③ Right prominence ATR: ____° |
| ****(3) Scoliometer Examination****  Was a scoliometer used? ① Yes(Proceed to scoliometer measurement below) ② No Scoliometer Readings: ① Thoracic ATR ____° ② Thoracolumbar ATR ____° ③ Lumbar ATR ____° |
| ****II. Sagittal Plane Deformity Screening(......)**** |
| ****III. Medical History**** |
| ****(6) History (multiple selections allowed)**** ① None ② Family history of spinal deformity ③ History of spinal trauma ④ History of spinal surgery Other special circumstances: __________________________________ |
| ****Screening Conclusion:**** ① Normal ② Postural imbalance ③ Scoliosis (Grade ____) ④ Abnormal Lordosis ⑤ Abnormal Kyphosis |
| ****Recommendation:**** _________________________________________________________________________ ****Signature of Examiner:**** __________________________________ |


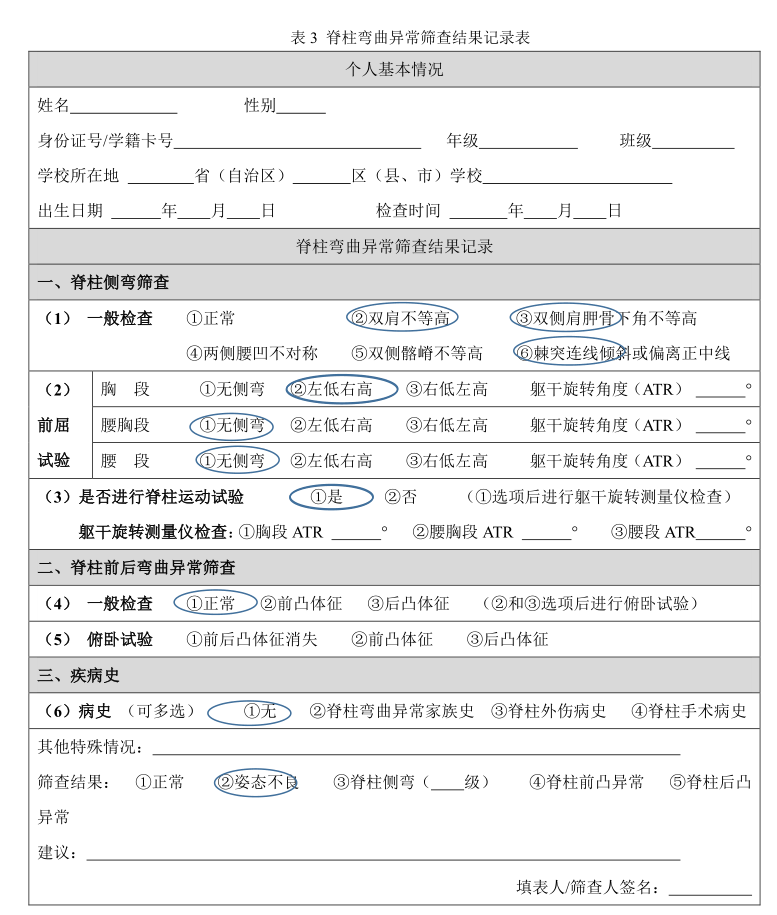

Supplement: Supplementary file 1 [file Table_1.docx]
